# Crystal structure of the capsular polysaccharide synthesizing protein CapE of *Staphylococcus aureus*

**DOI:** 10.1042/BSR20130017

**Published:** 2013-06-11

**Authors:** Takamitsu Miyafusa, Jose M. M. Caaveiro, Yoshikazu Tanaka, Martin E. Tanner, Kouhei Tsumoto

**Affiliations:** *Medical Proteomics Laboratory, Institute of Medical Science, The University of Tokyo, Minato-ku, Tokyo 108-8639, Japan; †Department of Medical Genome Sciences, School of Frontier Sciences, The University of Tokyo, Minato-ku, Tokyo 108-8639, Japan; ‡Department of Chemistry, University of British Columbia, 2036 Main Mall, Vancouver, British Columbia V6T 1Z1, Canada; §Department of Chemistry and Biotechnology, School of Engineering, The University of Tokyo, Tokyo 113-8656, Japan

**Keywords:** capsular polysaccharide, conformational change, pathogenic bacterium, SDR enzyme, UDP–sugar, X–ray crystallography, CP, capsular polysaccharide, hUDGH, human UDP-α-D-glucose dehydrogenase, SDR, short-chain dehydrogenase/reductase, SEC, size-exclusion chromatography, UDP-6N_3_-GlcNAc, 6′-Azido-6′-deoxy-UDP- N-acetylglucosamine

## Abstract

Enzymes synthesizing the bacterial CP (capsular polysaccharide) are attractive antimicrobial targets. However, we lack critical information about the structure and mechanism of many of them. In an effort to reduce that gap, we have determined three different crystal structures of the enzyme CapE of the human pathogen *Staphylococcus aureus*. The structure reveals that CapE is a member of the SDR (short-chain dehydrogenase/reductase) super-family of proteins. CapE assembles in a hexameric complex stabilized by three major contact surfaces between protein subunits. Turnover of substrate and/or coenzyme induces major conformational changes at the contact interface between protein subunits, and a displacement of the substrate-binding domain with respect to the Rossmann domain. A novel dynamic element that we called the latch is essential for remodelling of the protein–protein interface. Structural and primary sequence alignment identifies a group of SDR proteins involved in polysaccharide synthesis that share the two salient features of CapE: the mobile loop (latch) and a distinctive catalytic site (MxxxK). The relevance of these structural elements was evaluated by site-directed mutagenesis.

## INTRODUCTION

*Staphylococcus aureus* is normally a harmless commensal bacterium residing in 25% of the adult population, yet is a dangerous human pathogen in susceptible individuals [1,2]. *S. aureus* is also notorious for its capacity to develop resistance against antibiotics. For example, methicillin- and vancomycin-resistant strains of *S. aureus* are among the major causes of nocosomial and community infections in the USA [3,4]. We are in urgent need of new targets and innovative strategies to effectively combat *S. aureus* and other dangerous pathogens [5].

The biosynthetic machinery that generates the CP (capsular polysaccharide) is absent in humans, and represents an attractive target to fight *S. aureus* [6–8]. CP forms a thick layer of carbohydrate on the cell surface conferring anti-phagocytic properties, and helping *S. aureus* to persist in the bloodstream of the infected host. More than 70% of clinical isolates of *S. aureus* belong to either the CP5 or the CP8 serotypes [8,9].

The basic structure of CP of serotypes CP5 and CP8 consists of alternating units of three types of monosaccharides: *N*-acetyl-L-fucosamine, *N*-acetyl-D-fucosamine, and *N*-acetyl-D-mannosamine uronic acid. The UDP-L-FucNAc (UDP-activated form of *N*-acetyl-L-fucosamine) is synthesized from its glucosamine precursor (UDP-D-GlcNAc). This reaction has been described in *S. aureus* and several other pathogenic bacteria possessing CP such as *Streptococcus pneumoniae* and *Bacteroides fragilis* [10,11]. L-FucNAc is also utilized in the synthesis of the lipopolysaccharide of some Gram-negative bacteria [12,13].

**Figure 1:**
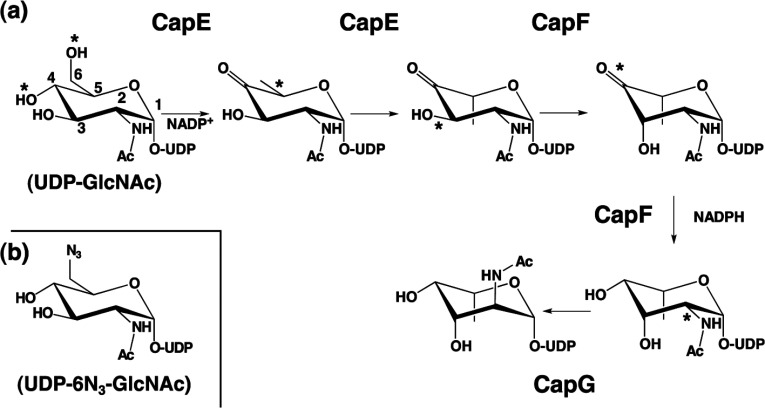
Biosynthetic pathway of UDP-L-FucNAc in *S. aureus* (**a**) Synthesis of UDP-L-FucNAc requires the sequential activity of enzymes CapE, CapF and CapG. The enzyme CapE is a bifunctional enzyme catalysing the 4,6-dehydration of UDP-D-GlcNAc and a subsequently C5-epimerization. CapF catalyses the C3-epimerization of the previous intermediate, followed by the reduction of the keto-sugar at position C4. CapG catalyses a C2-epimerization to yield the final product UDP-L-FucNAc. The asterisks indicate the chemical groups subjected to enzymatic modification. This pathway is adapted from previous mechanistic studies [12,14]. (**b**) Structure of the substrate analogue UDP-6N_3_-GlcNAc, where the hydroxyl group at C6 of the substrate is replaced by an azide substituent [20].

The transformation of UDP-D-GlcNAc in UDP-L-FucNAc requires three enzymes (CapE, CapF and CapG) in *S. aureus* [12,14]. These three enzymes catalyse a total of five chemical reactions (Figure 1). The recently determined crystal structure of CapF unveiled a unique architecture composed of two distinctive domains [15]: a N-terminal domain belonging to the SDR (short-chain dehydrogenase/reductase) superfamily of proteins [16], and a C-terminal domain displaying a standard cupin fold [17]. A previous study of the homologous protein WbjB, together with the structural study of the enzyme CapF (just downstream of CapE), indicates that CapE is a bi-functional enzyme [14,15]. The two reactions catalysed by CapE are the C-4/C-6 dehydration and the C-5 epimerization of the UDP–sugar. The enzyme FlaA1 from *Helicobacter pylori* displays 40% homology to CapE and catalyses the same enzymatic reactions [18,19]. However, the key catalytic tyrosine residue of FlaA1 is replaced with methionine in CapE, suggesting mechanistic differences between them.

Herein we present the first crystal structure of CapE, revealing that this enzyme forms a stable and functional homo-hexamer. From the comparison of three crystal structures, we identified a novel and dynamic element unique to the capsular-polysaccharide synthesizing proteins that we called the latch. The special configuration of the active site of CapE, together with the unique features of the latch, exposed a distinctive group of enzymes of bacterial origin within the SDR superfamily sharing these two special elements.

## EXPERIMENTAL

### Substrate analogue

The synthesis of UDP-6N_3_-GlcNAc (6′-Azido-6′-deoxy-UDP-N-acetylglucosamine) was carried out as previously described [20].

### Protein expression and purification

CapE with a His_6_ tag at the N-terminus was expressed in *Escherichia coli* BL21 (DE3) cells, and purified as described previously [15,21]. After SEC (size-exclusion chromatography), fractions of CapE were dialysed in 10 mM Tris/HCl (pH 9.0), 30 mM NaCl and 1 mM DTT (dithiothreitol), and concentrated with a 100 kDa Centriprep filtration unit (Millipore) prior to crystallization. For activity assays, protein fractions were stored at −20°C in a solution supplemented with 40% (v/v) glycerol. Protein concentration was determined spectrophotometrically at 280 nm using the calculated molar extinction coefficient of the protein (*ϵ*=19200 M^−1^ cm^−1^). Muteins of CapE were prepared with a Quick-Change kit following the instructions of the manufacturer. Protein expression and purification were carried out as above.

### Protein crystallization

Crystals of CapE suitable for X-ray diffraction analysis were obtained by the hanging drop method by mixing 1 μl of fresh protein solution at 8 mg/ml and 1 μl of crystallization solution. Crystals of wild-type CapE with coenzyme bound were obtained in 0.72 M sodium succinate (pH 7.0) and 1.4 M potassium formate. Rod-shaped crystals grew to an approximate size of 30×30×100 μm^3^ within 1 week. Crystals were transferred to a cryoprotectant solution consisting of mother liquor supplemented with 25% (v/v) glycerol, plunged into liquid N_2_ and stored until data collection.

Crystals of CapE with substrate analogue were obtained by the co-crystallization method using 100 μM UDP-6N_3_-GlcNAc. Extensive screening with an Oryx8 robot (Douglas Instruments) [22] yielded a suitable solution composed of 100 mM Hepes/NaOH (pH 7.5) and 1.5 M Li_2_SO_4_. For freezing, single crystals were passed through a small drop of paratone (Hampton Research) and plunged in liquid N_2_. Crystals of mutein K126E were obtained in 100 mM Hepes (pH 7.5), 2% (v/v) PEG400 and 2.0 M ammonium sulfate. Suitable crystals were soaked in mother liquor supplemented with 25% (v/v) glycerol and 500 μM substrate (the substrate was not observed in the electron density) and stored in liquid N_2_.

### Data collection and refinement

**Figure 2:**
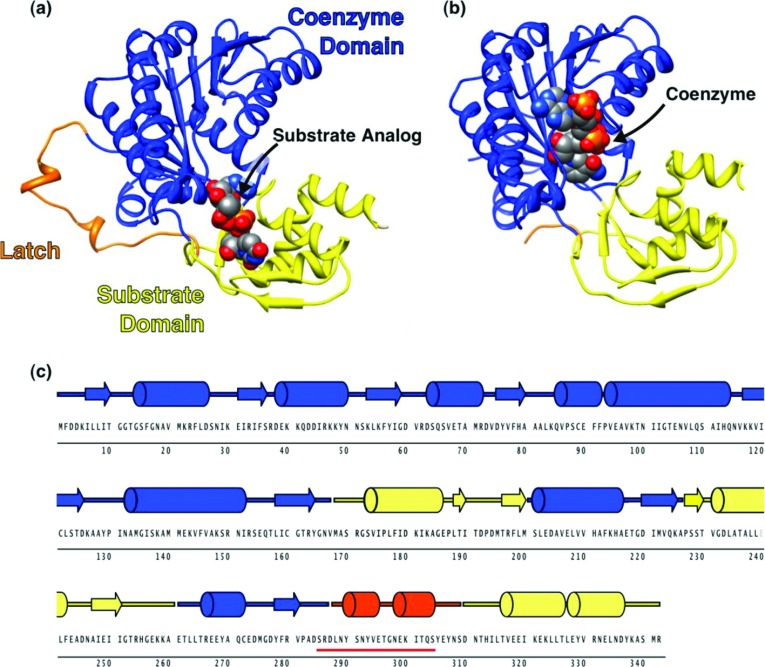
Crystal structure of CapE Structure of (**a**) CapE with substrate analogue UDP-6N_3_-GlcNAc bound, and (**b**) K126E mutein with coenzyme NADPH bound. The coenzyme-binding domain, substrate-binding domain, and latch are coloured in blue, yellow and orange, respectively. The ligands are depicted as spheres with CPK colours. The figure was prepared with CHIMERA [50]. (**c**) Primary and secondary structure of CapE. The underlined sequence (red line) corresponds to the disordered region in K126E.

Suitable crystals of CapE were mounted under a stream of cold nitrogen (100 K) at beamlines BL5A and AR-NE3A of the Photon Factory (Tsukuba, Japan). Data were processed with the program MOSFLM [23] and merged and scaled using the program SCALA of the CCP4 program suite [24]. The structure of mutein K126E was determined by the method of molecular replacement with the program PHASER [25,26] using the coordinates of FlaA1 of *H. pylori* (PDB entry code 2gn4) as the search model. Coordinate refinement was carried out with PHENIX [27], REFMAC5 [28] and COOT [29]. Data were further refined with REFMAC5 using TLS parameterization. Each protomer was split in the three groups shown in Figure 2 of the Results and Discussion section [30]. The structure of the apo-CapE and apo-K126E were determined by molecular replacement using the structure of holo-CapE. Refinement was performed as above. Model quality was assessed with PROCHECK [31]. Data collection and refinement statistics are summarized in Table 1.

**Table 1:** Data collection and refinement statistics Values in parenthesis correspond to the highest resolution bin.

	Wild-type		K126E
Properties	UDP-6N_3_-GlcNAc	NADPH	NADPH
Data collection			
Space group	*P*321	P6_3_22	H3_2_
Unit cell			
Dimensions (Å)	a=b=123.78, c=104.46	a=b=125.18, c=101.79	a=b=160.5, c=80.05
Angles (°)	α=β=90, γ=120	α=β=90, γ=120	α=β=90, γ=120
Wavelength (Å)	1.0000	1.0000	1.0000
Resolution range (Å)	34.8−2.10	34.05−2.80	40.13−2.20
Total observations	291899	103149	93553
Unique observations	54166	12071	20104
*I*/*σ* (*I*)	11.0 (4.1)	12.2 (2.8)	8.9 (2.5)
Completeness (%)	99.9 (100)	99.9 (100)	99.9 (100)
*R*_merge_ (%)*	10.4 (33.8)	11.9 (79.8)	8.8 (49.8)
Multiplicity	5.4 (5.2)	8.5 (8.9)	4.7 (4.4)
Refinement			
*R*_work_*/R*_free_ (%)†	16.4/20.7	23.8/27.6	19.6/26.4
Number of protein chains	2	1	1
Number of protein residues	693	262	314
Number of protein atoms	5562	2137	2504
Number of ligands	18‡	1	6
Number of ligand atoms	168	48	73
Number of water molecules	385	3	82
B-factor, protein (Å^2^)	29.0	81.9	50.1
B-factor, ligands (Å^2^)	53.3	80.4	76.8
B-factor, water (Å^2^)	36.0	49.2	46.5
RMSD bonds (Å)	0.023	0.008	0.017
RMSD bonds (°)	2.19	1.363	2.01
Co-ordinate error (Å)	0.14	0.62	0.24
Ramachandran plot			
Preferred regions (%)	97.5	92.7	95.9
Allowed regions (%)	2.2	7.3	4.1
Outliers (%)	0.3	0.0	0.0
PDB codes	3W1V	3VVB	3VVC

*R_merge_=Σ_hkl_ Σ*_i_*|*I*(hkl)*_i_*−[*I*(hkl)]|/Σ_hkl_ Σ*_i_I*(hkl).

†*R*_work_=Σ_hkl_ |*F*(hkl)*_o_*−[*F*(hkl)_c_]|/Σ_hkl_*F*(hkl)_o_; *R*_free_ was calculated as *R*_work_, where *F*(hkl)_o_ values were taken from 5% of data not included in the refinement.

‡This structure contains 14 sulfate ions from the crystallization solution.

### Enzymatic assay

Enzymatic activity of CapE was monitored by the method described in Miyafusa et al. [15]. In a typical assay, substrate UDP-D-GlcNAc (Wako) at 200 μM was mixed with 2 μM CapE. The assay buffer was composed of 20 mM Tris/HCl (pH 8.0). Total volume was 100 μl. Assay mixtures were incubated at 37°C for 2 h, after which the reaction was stopped by addition of 100 μl of ice-cold phenol/chloroform/isoamyl alcohol at a 25:24:1 molar ratio. The supernatant containing the sugars were mixed with 100 μl of chloroform and analysed by HPLC using a CarboPac PA1 anion-exchange column (Dionex) as described previously [32]. The overall conversion to products was calculated from the consumption of substrate UDP-D-GlcNAc.

### Differential scanning calorimetry

The thermal stability of CapE was determined in a VP-capillary microcalorimeter (GE Healthcare). Proteins at a concentration of 18 μM were equilibrated in a solution containing 50 mM Hepes (pH 7.4) and 150 mM NaCl. Thermograms were recorded between 283 K and 373 K at a rate of 1 K min^−1^. The buffer baseline was subtracted from the protein thermogram and the data subsequently were normalized by protein concentration and adjusted to a two-stage unfolding curve [33] using the program ORIGIN supplied by the manufacturer.

## RESULTS AND DISCUSSION

### Crystal structure of CapE

We determined three crystals structures of CapE by X-ray diffraction methods at resolutions 2.1–2.8 Å (Table 1). Two structures correspond to the binary complex of the enzyme with coenzyme (wild-type protein and inactive mutein K126E). The third structure corresponds to wild-type protein (coenzyme-free) in complex with the substrate analogue UDP-6N_3_-GlcNAc.

A three-dimensional homology search in the DALI server [34] indicates that CapE is a member of the extended SDR superfamily [35]. As often seen in this group of enzymes, CapE shares little or very little similarity at the primary sequence level with the other members of this family (10–25% identity). The only exception is FlaA1 of *Helicobacter pylori* (40% identity), which is also the closest structural homolog of CapE in the protein data bank (PDB entry code 2gn4; rmsd=2.0±0.1 Å). We note that the enzyme following CapE in the biosynthetic route of UDP-D-FucNAc in *S. aureus*, CapF, has the lowest similarity score among the first 950 structural hits found with the program DALI [34].

CapE consists of three well-defined regions: a Rossmann domain for binding the coenzyme NADPH, a substrate binding domain, and a 23-residue loop peripheral to the protein core (Figures 2A and 2B). The first two regions are well-conserved elements of the SDR family of enzymes [35]. Segments corresponding to each domain are interspersed along the primary sequence of the enzyme (Figure 2C). The loop comprising residues 287–309 constitutes a novel structural element in this family of proteins. We called this loop the *latch* because it engages two molecules of CapE in the crystallographic dimer. The latch is not observed in the two crystal structures of CapE with coenzyme bound because of dynamic disorder. In contrast, the latch is clearly observed in the structure with substrate analogue bound (see below). We note that the tertiary structure of CapE and FlaA1 differs significantly from each other at the latch region (Supplementary Figure S1 at http://www.bioscirep.org/bsr/033/bsr033e043add.htm). Whereas the latch connects two protomers of CapE of the hexameric complex, the equivalent loop of FlaA1 (residues 292–318) folds into its own protein chain.

**Figure 3:**
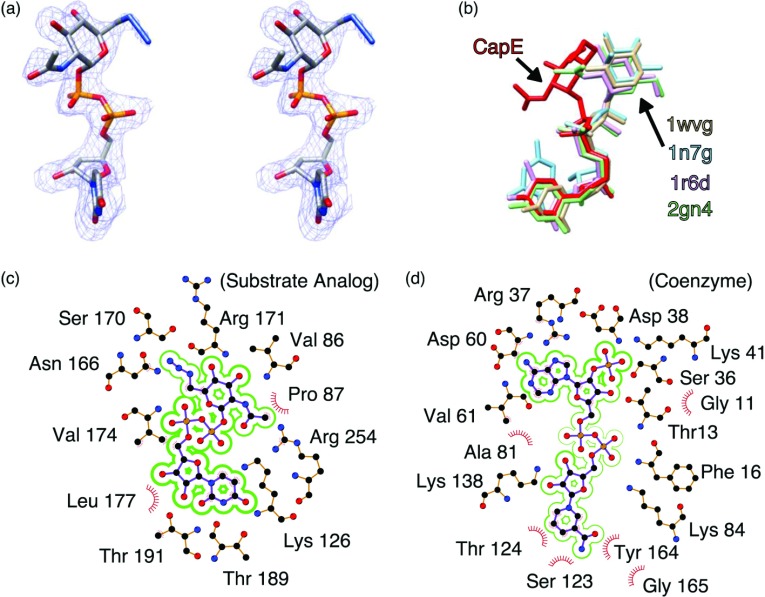
Conformation of substrate analogue and coenzyme (**a**) Stereoview of the substrate analogue UDP-6N_3_-GlcNAc. The electron density correspond the sigma-A weighted 2Fo–Fc map contoured at 1.0 σ. The substrate analogue is depicted with CPK colours. (**b**) Conformation of various nucleotide-sugars bound to representative SDR enzymes. The panel depicts CapE (this study, red); CDP-β-D-xylose bound to CDP-D-glucose 4,6-dehydratase (1wvg, yellow); GDP-rhamnose bound to GDP-mannose 4,6-dehydratase (1n7g, cyan); dTDP-D-glucose bound to dTDP-glucose 4,6-dehydratase (D128N/E129Q mutein, 1r6d, pink); and UDP-GlcNAc bound to FlaA1 (UDP-GlcNAc inverting 4,6-dehydratase, 2gn4, green). (**c**) Residue environment around the substrate analogue in wild-type CapE and (**d**) in the binding pocket of the coenzyme in K126E. Panels (**c**) and (**d**) were generated with the program LIGPLOT [51].

The substrate-binding domain consists of residues 168–199, 227–260 and 310–337. The substrate-analogue UDP-6N_3_-GlcNAc is clearly visible in the electron density map (Figure 3A). The electron density of the azide moiety is weaker than that in other sections of the substrate analogue, probably because of radiation damage at this functional group. Although the sugar ring of the substrate analogue protrudes into the coenzyme domain (where the active site is located), it occupies a non-catalytic conformation compared with that of other representative SDR enzymes (Figure 3B). The conformation of UDP-6N_3_-GlcNAc bound to CapE is similar to that of the inhibitor UDP-D-GalNAc bound to the homologous enzyme FlaA1 [18] (Supplementary Figure S1). The substrate analogue engages residues of the binding pocket through multiple polar interactions (Figure 3C and Supplementary Table S1 at http://www.bioscirep.org/bsr/033/bsr033e043add.htm). In particular, the UDP moiety of the substrate analogue establishes numerous H-bond and electrostatic interactions with polar residues of the protein.

The coenzyme-binding region is the largest functional element of CapE. This domain adopts a classical Rossmann fold [16,35], comprising residues 1–167, 200–226 and 261–286. Although NADPH was not added during protein purification, or during the crystallization trials, it is clearly visible in the crystal structure of wild-type and K126E, suggesting that the cofactor binds the enzyme tightly (Supplementary Figure S2 at http://www.bioscirep.org/bsr/033/bsr033e043add.htm). A detailed view of the local environment around the coenzyme is depicted in Figure 3(D). The coenzyme engages in a dense network of non-covalent interactions with the enzyme, including polar and van der Waals forces, and hydrophobic interactions. Unexpectedly, the coenzyme moiety is not observed in the structure of CapE in complex with substrate analogue. This is surprising because the superposition of the two types of CapE complexes does not suggest direct clashes between the substrate analogue and the coenzyme moiety. It is possible that the departure of the coenzyme is stimulated by a kinetic mechanism similar to that of SDR proteins ArnA and UDP-xylose synthase [36]. Alternatively, it is conceivable that the substrate analogue binds to CapE in a non-native conformation that is incompatible with the binding of the coenzyme.

### CapE is a functional hexamer

**Figure 4:**
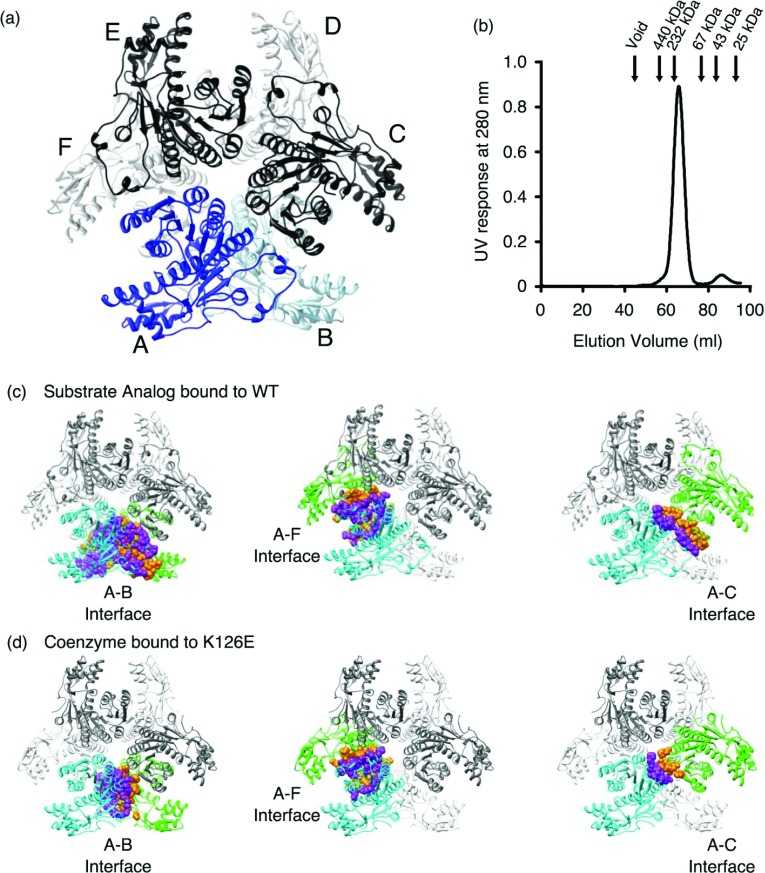
Hexameric organization of CapE (**a**) CapE forms a hexamer organized as a trimer of dimers (3×2). The structure corresponds to wild-type CapE in complex with substrate analogue (ligand is not shown). The protein subunits of one of the pseudo-dimers are depicted in dark and light blue (chains A and B). The other subunits are depicted in black and grey. (**b**) SEC profile of CapE. The arrows indicate the elution of the calibration standards. CapE elutes with an apparent molecular weight of 210 kDa. A minor fraction of monomer (8%) with an apparent molecular weight of 40 kDa is also observed. The predicted molecular weight of one subunit of CapE is 39 kDa, and that of the hexamer is 230 kDa. The peak corresponding to the dimer was not observed. (**c**) Interaction surfaces between protein subunits. The three main contact interfaces correspond to subunits A–B, A–F and A–C. (**d**) Same interaction surfaces in mutein K126E.

Analysis of the crystallographic symmetry reveals that CapE is a homo-hexamer in the crystal form (Figure 4). The protein assembles as a trimer of dimers (3×2). The hexameric organization of CapE constitutes a rare example within the SDR family. This organization is only shared by FlaA1, which is the SDR enzyme with the highest structural homology to CapE [18]. SEC indicates that the hexameric complex is the predominant species in solution (Figure 4B). The hexamer coexists with a small fraction of monomer (~8%), but not dimer. Moreover, CapE undergoes two-state thermal unfolding without intermediates, suggesting that the hexamer is the most stable form of CapE in solution (Supplementary Figure S3 at http://www.bioscirep.org/bsr/033/bsr033e043add.htm) [37].

The evaluation of the contact interface with the PISA server [38] indicates that the hexamer of CapE (with substrate analogue bound) buries nearly 24000 Å^2^ of surface area upon oligomerization, i.e. 24% of the total solvent-exposed surface. Each protomer of CapE interacts with adjacent molecules through three major interfaces (Figure 4C). The largest interface, between molecules A and B, buries 5016 Å^2^ and involves 122 residues (28% of total) (Table 2). This interface corresponds to the typical dimerization interface observed in numerous SDR enzymes [39,40]. The interfaces A–F and A–C are smaller, burying 2044 Å^2^ and 934 Å^2^, respectively.

**Table 2:** Interaction surface area between CapE protomers

Interface*	Protein	Buried area (Å^2^)	Number of residues†	No. H-Bond
A–B	WT (UDP-6N_3_-GlcNAc)	5016	122	28
	WT (NADPH)	2162	58	4
	K126E (NADPH)	1988	58	4
A–F	WT (UDP-6N_3_-GlcNAc)	2044	58	12
	WT (NADPH)	2066	58	10
	K126E (NADPH)	2070	58	12
A–C	WT (UDP-6N_3_-GlcNAc)	934	32	6
	WT (NADPH)	346	14	0
	K126E (NADPH)	334	14	0
Total	WT (UDP-6N_3_-GlcNAc)	7994	212	46
	WT (NADPH)	4574	130	14
	K126E (NADPH)	4392	130	16

*Protomers of CapE as shown in Figure 4(A).

†Some residues are counted more than once because they participate in more than one surface.

The A–B and A–C surface areas of CapE in complex with coenzyme are significantly smaller than that of CapE with substrate analogue bound. The A–B and A–C surface areas calculated from the coordinates of CapE with coenzyme bound are reduced by 60 and 65%, respectively (Figure 4C, Table 2). The reason is the dynamic disorder occurring at these two contact regions. In fact, the number of residues observed at these two interfaces is greatly diminished (Table 2). On the contrary, the A–F interaction surface remains essentially unchanged regardless of the ligand present in the crystal. The surface area, number of residues and number of non-covalent interactions in the A–F region remains constant among the three crystals structures (Supplementary Table S2 at http://www.bioscirep.org/bsr/033/bsr033e043add.htm). hUDGH (human UDP-α-D-glucose dehydrogenase) is a well-studied hexameric enzyme showing large structural changes during its catalytic cycle [41,42]. The hexamer-building interface of hUDGH is quite flexible and therefore the hexamer readily dissociates into dimers and tetramers. On the contrary, the hexamer of CapE seems more stable and remains together even after the disruptive effects described above, or after large conformational changes (see below). We cannot rule out that some of the conformational changes are influenced by crystal packing forces, and therefore may not be of functional significance.

### Conformational changes

**Figure 5:**
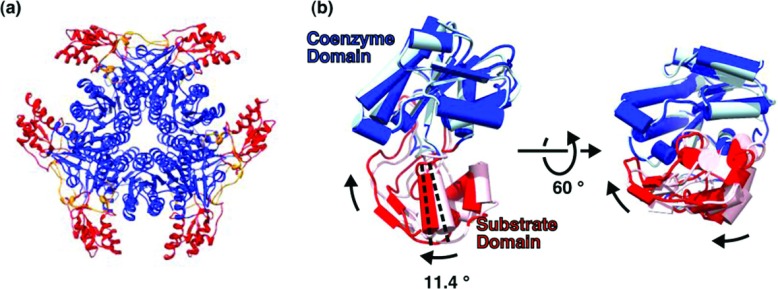
Conformational changes in the substrate-binding domain (**a**) Topology of the hexamer. The coenzyme-binding domain (blue) and the substrate-binding domain (red) occupy the central and peripheral regions of the complex, respectively. The latch (orange) is located in an intermediate position. (**b**) Conformational change. Binding of the substrate analogue, and loss of the coenzyme, induces a rotation of 11° and a displacement of the substrate domain towards the Rossmann domain. Dark and light colours correspond to wild-type protein and K126E, respectively. The rotation angle was calculated with the program DYNDOM [43].

The Rossmann domain of CapE is located at the centre of the hexamer and mediates most of the inter-chain contacts, whereas the substrate-binding domain is found at the periphery of the complex (Figure 5). This arrangement could facilitate conformational changes during the catalytic cycle at the substrate-binding domain, since this domain is more exposed to the solvent.

The crystals structures of CapE with analogue bound or with coenzyme bound were compared with the program DYNDOM [43] to quantify their conformational changes. The structure of K126E with coenzyme bound was chosen over the equivalent structure of wild-type CapE because of the better resolution achieved with the mutated protein (Table 1). The analysis with DYNDOM indicates that the substrate-binding domain rotates 11° and moves a few angstroms towards the coenzyme-binding domain upon exit of the coenzyme and binding of substrate analogue (Figure 5B). The largest shift occurs at residues Gly^256^ and Gly^252^ (6.0 Å). Although these observations must be understood within the context of the crystal structure, they are suggestive of a significant mobility at the substrate domain during catalysis.

**Figure 6:**
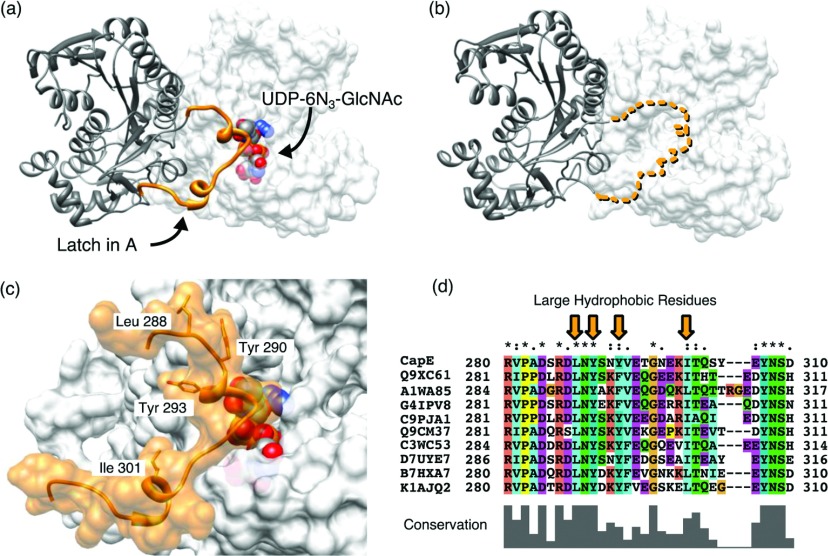
Analysis of the latch (**a**) Structure of the latch at the A–B dimer interface. The latch (orange) of subunit A (grey ribbons) associates with the substrate-binding domain of molecule B (grey surface). The substrate analogue is depicted only in subunit B (CPK representation). (**b**) Same view of K126E. The latch is not modelled because of disorder. The dotted line represents the hypothetical position of the latch in the same position as shown in panel (**a**). (**c**) Close view of the latch of subunit A interacting with the substrate-binding pocket of subunit B. The hydrophobic residues Leu^288^, Tyr^290^, Tyr^293^ and Ile^301^ fit on the groves of subunit B with high surface complementarity. (**d**) Sequence alignment of the residues belonging to the latch. The panel shows the top-ten solutions found by BLAST [52]. The sequences belong to various genus of Gram-positive and Gram-negative pathogenic bacteria (from top to bottom: *Staphylococcus*, *Pseudomonas*, *Acidovorax*, *Hyphomicrobium*, *Vibrio*, *Pasteurella*, *Fusobacterium*, *Listeria*, *Bacillus* and *Enterococcus*). Each sequence is identified by their accession code in the UNIPROT database.

Another important dynamic element is the latch (residues 287–309, Figure 6). The latch occupies a position between that of the substrate-binding and Rossmann domains (Figure 5A). The latch is disordered in the two crystal structures with coenzyme bound (Figure 6B and Supplementary Figure S4 at http://www.bioscirep.org/bsr/033/bsr033e043add.htm). The latch connects two contiguous subunits of CapE, and forms a large fraction of the A–B and A–C interface of the hexamer with very high shape complementarity (*S*_C_=0.79 [44]) (Figure 6C and Supplementary Figure S5 at http://www.bioscirep.org/bsr/033/bsr033e043add.htm). Together with the residues of neighbouring subunits of CapE, the latch contributes >3500 Å^2^ of buried surface area, including 28 H-bonds. The interaction between the latch and the adjacent protomer is also held together by contacts involving four large hydrophobic residues: Leu^288^, Tyr^290^, Tyr^293^ and Ile^301^. In addition, the latch occupies a strategic position at the entrance of the substrate-binding pocket, potentially regulating the access of the substrate to the active site (Figure 6C).

A search of tertiary structure homologues of the latch with the DALI server [34] did not find any example of this structural element in the protein data base. For example, the SDR enzyme FlaA1 (the closest structural homologue of CapE) also forms a hexamer of similar size to that of CapE, but do not exhibit this element (Supplementary Figure S1) [18]. On the contrary, the program Protein BLAST indicates that the primary sequence of the latch is well conserved among a variety of Gram-positive and Gram-negative bacteria of pathogenic potential (Figure 6D). The genera of these pathogenic bacteria are *Pseudomonas*, *Acidovorax*, *Hyphomicrobium*, *Vibrio*, *Pasteurella*, *Fusobacterium*, *Listeria*, *Bacillus* and *Enterococcus* (in addition to *Staphylococcus*). Serotypes of a majority of these bacteria are encapsulated, suggesting a direct association between this novel group of SDR enzymes, the production of CP and pathogenesis [6,12,45–49].

### Site-directed mutagenesis supports the structural findings

The coenzyme-binding domain hosts the canonical catalytic triad, which invariably contains the sequence YxxxK in SDR enzymes (except for a minor group of divergent SDR enzymes, which are characterized by a YxxMxxxK motif). Strikingly, the corresponding sequence of CapE is altered to M^134^xxxK^138^ without the catalytic tyrosine. Importantly, the sequence alignment of the family of proteins sharing the latch (see Figure 6D) demonstrates an absolute conservation of the catalytic residues of CapE (Supplementary Figure S6 at http://www.bioscirep.org/bsr/033/bsr033e043add.htm). In contrast, the other SDR structural homologues do invariably possess the canonical YxxxK motif (Supplementary Figure S7 at http://www.bioscirep.org/bsr/033/bsr033e043add.htm). We propose that the unique structural element that we called the latch, and the unique composition of the active site residues define a novel subfamily of polysaccharide-synthesizing enzymes within the SDR super-family of proteins.

**Figure 7:**
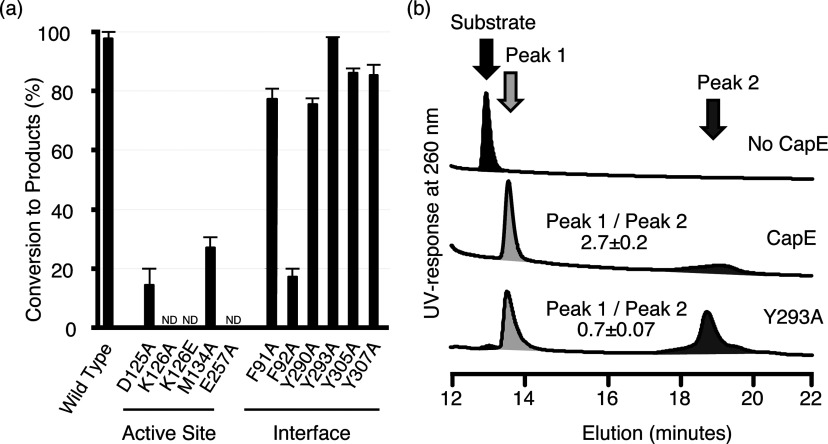
Mutational analysis (**a**) Wild-type CapE or muteins (2 μM) were incubated with UDP-D-GlcNAc (200 μM) for 2 h at 37°C. Consumption of substrate UDP-D-GlcNAc was monitored by HPLC as described in experimental procedures. The bars correspond to the average of three independent assays ± S.D. (**b**) Representative HPLC profiles. The substrate UDP-D-GlcNAc was incubated with no CapE (top), with wild-type CapE (middle) and with mutein Y293A (bottom).

To strengthen the structural analysis we prepared several muteins of CapE by site-directed mutagenesis (Figure 7 and Supplementary Table S3 at http://www.bioscirep.org/bsr/033/bsr033e043add.htm). No excess coenzyme was added in the enzymatic assay because CapE was purified in complex with the coenzyme. The level of NADPH bound to CapE was essentially constant among all muteins as estimated from the ratio Abs260/Abs280 (Supplementary Table S3). Firstly, individual residues of the active site and substrate-binding pocket were mutated: Asp^125^, Lys^126^, Met^134^ and Glu^257^ were changed to Ala (Lys^126^ was also mutated to Glu and its crystal structure discussed above). All these individual muteins exhibited a much-diminished activity compared with the wild-type enzyme. The muteins K126A, K126E and E257A where completely inactive, demonstrating the importance of these residues for the functional binding of the substrate. Meanwhile the activity of D125A and M134A was strongly impaired (4–6-fold lower conversion than WT protein). We note that all the muteins eluted as hexamers during their final step of purification by SEC.

Secondly, we evaluated the properties of the A–B surface (essentially the latch) by mutating separately five different residues to Ala: Phe^91^, Tyr^290^, Tyr^293^, Tyr^305^ and Tyr^307^. The last four muteins belong to the latch region. The mutations did not alter significantly the levels of conversion compared with the wild-type enzyme (Figure 7A). However, although the values of conversion of Y293A and wild-type protein are identical, the ratio of their enzymatic products (computed from the area under the HPLC peaks) is inverted in Y293A in comparison with wild-type CapE. The relative areas of peak-1 with respect to peak-2 in wild-type or in mutein Y293A were 2.7 and 0.7, respectively (Figure 7B). Similarly, the selectivity is inverted in all muteins belonging to the latch, but not in F91A, a residue not belonging to the latch (Supplementary Table S3). Changes in the relative abundance of intermediates do not necessarily reflect a change of their thermodynamic equilibrium, but probably a slow down of their rate of interconversion. Our next challenge will consist in the identification of these unstable intermediates, which will allow an in-depth discussion of the structure/function relationship and catalytic mechanism of CapE. We note that in contrast to the results obtained with the muteins belonging to the interface A–B, the mutein F92A of the A–F interface led to a much-reduced activity (<20% conversion).

### Conclusion

We determined the first set of crystal structures of the enzyme CapE of *S. aureus* belonging to the biosynthetic pathway of CP. CapE forms a robust hexamer held together by three different interfaces, A–B, A–F and A–C. The largest A–B interface contains a mobile motif that we denominated the latch. The latch is a flexible element that increases the contact surface area of adjacent monomers of CapE in the presence of the substrate analogue UDP-6N_3_-GlcNAc. The structural analysis also demonstrates a rotation of the substrate-binding domain when the substrate analogue binds to the enzyme. Importantly a sequence alignment of the latch, and of the active site residues of CapE, has identified a novel subfamily of SDR enzymes involved in the synthesis of polysaccharide of Gram-positive and Gram-negative pathogenic bacteria.
